# Pleuroparenchymal abnormalities in cancer patients undergoing CTPA for suspected pulmonary embolism

**DOI:** 10.3389/fradi.2026.1901919

**Published:** 2026-10-13

**Authors:** Mohammad F. Allahham, Haya Mustafa, Jana Faroun, Alhareth M. Amro, Fuad M. Allahham, Oadi N. Shrateh, David Murphy

**Affiliations:** 1 Faculty of Medicine, Al-Quds University, Jerusalem, Palestine; 2 Head of the Department of Anesthesia, Augusta Victoria Hospital, Jerusalem, Palestine; 3 Department of Radiology, St. Vincent’s University Hospital, Dublin, Ireland

**Keywords:** cancer, computed tomographic pulmonary angiography (CTPA), lung, pulmonary embolism (PE), pulmonary infarction

## Abstract

**Objectives:**

To characterize pleuroparenchymal abnormalities identified on computed tomography pulmonary angiography (CTPA) in cancer patients investigated for suspected pulmonary embolism (PE) and to compare imaging findings between patients with and without confirmed PE.

**Methods:**

This single-center retrospective observational study included 113 consecutive cancer patients who underwent CTPA for suspected acute PE between January 2022 and January 2026. Radiology reports and electronic medical records were reviewed for demographic data, malignancy type and stage, PE-related imaging characteristics, and pleural and pulmonary parenchymal abnormalities. Imaging findings were compared between PE-positive and PE-negative groups. An exploratory multivariable model with a restricted set of imaging predictors was constructed to limit overfitting, with correction for multiple comparisons and bootstrap-based internal validation. Exploratory subgroup analyses by malignancy type and disease burden were also performed.

**Results:**

PE was confirmed in 32 patients (28.3%). The median age was 59 years (IQR, 51–65), and 42.5% were female. The highest PE prevalence was observed in gastrointestinal, gynecologic, and thoracic malignancies. At nominal testing, infarct-like wedge-shaped opacity was more frequent in PE-positive patients (12.5% vs. 1.2%; OR, 11.43; *p* = 0.022), whereas ground-glass opacity (15.6% vs. 35.8%; OR, 0.33; *p* = 0.041) and interstitial/septal change (3.1% vs. 25.9%; OR, 0.09; *p* = 0.007) were less frequent. None remained statistically significant after false-discovery-rate correction (*q* = 0.133, 0.166, and 0.082, respectively). The parsimonious model had an apparent AUROC of 0.707 and an optimism-adjusted AUROC of 0.699, with acceptable grouped calibration (Hosmer–Lemeshow *p* = 0.522). No exploratory cancer-subgroup comparison met *q* < 0.05.

**Conclusions:**

Pleuroparenchymal abnormalities are highly prevalent in cancer patients undergoing CTPA for suspected PE; however, most findings are nonspecific. Infarct-like wedge-shaped opacity generated the strongest exploratory PE-associated signal, but no individual imaging comparison survived multiplicity correction and model estimates remained imprecise. These findings should therefore be considered hypothesis-generating and require validation in larger cohorts.

## Introduction

Pulmonary embolism, or PE, is a potentially fatal disorder and is one of the top three leading causes of cardiovascular mortality worldwide; coronary artery disease and strokes are the other two (1). PE is the most serious manifestation of venous thromboembolism (VTE) and usually arises from deep vein thrombosis (DVT) of the lower extremities, from which thrombi may detach and migrate through the venous circulation to occlude the pulmonary vasculature (2, 3). PE is estimated to occur in about 1 out of every 1,000 people per year, and the rate increases significantly with age to more than 11 per 1,000 among people aged 80 and over (4). PE causes much morbidity and mortality even though we have made advances in how to diagnose and treat it. In cases where PE is not treated, the mortality rate is nearly 30%; however, timely diagnosis and appropriate anticoagulation reduce PE-related mortality to approximately 8% (3, 4). Diagnosis remains a clinical challenge, partly because the signs and symptoms of PE vary widely among individuals, and many of its symptoms, including dyspnea, chest pain, and hemoptysis, overlap with other pulmonary and/or cardiac diseases (5, 6).

PE pathophysiology is classically described by Virchow's triad as being caused by venous stasis, endothelial injury, and hypercoagulability (7). The development of PE is influenced by many different factors, but malignancy represents one of the most clear-cut, potent risk factors for having a thromboembolic event that is many times greater than would occur in an individual without malignancy (8, 9). The hypercoagulable state associated with cancer is believed to develop through multiple pathways, including the production of procoagulant factors from the tumor cells itself, the presence of the inflammatory process driven by cytokines, thrombosis related to injury to the endothelium, and exposure to the effects of chemotherapy, surgery, indwelling catheters, and prolonged periods of immobility (9, 10). Consequently, PE represents a major cause of morbidity and mortality in cancer patients and significantly impacts the clinical course of oncology patients.

Once a thrombus occludes the pulmonary arterial bed, it results in an increase in pulmonary vascular resistance, right ventricular afterload, and decreased ability to exchange gases (11). These events may lead to right ventricular dysfunction, ventilation/perfusion (V/Q) mismatch, hypoxemia, and, in severe cases, hemodynamic instability or sudden death. Additional effects downstream, such as pulmonary infarction, hemorrhage, atelectasis, and inflammatory response, contribute to various pleuro-parenchymal abnormalities on imaging the chest with thoracic imaging (11, 12).

With excellent sensitivity and specificity in detecting intraluminal thrombus within the pulmonary arterial system, computed tomography pulmonary angiography (CTPA) has established itself as the imaging modality of choice for evaluating suspected PE (6, 13). It has become widely used and adopted as the initial diagnostic modality in most clinical settings due to its short acquisition time, availability, and noninvasive nature. CTPA has an advantage over other imaging modalities such as V/Q scintigraphy or conventional pulmonary angiography in that it allows for detailed three-dimensional assessment of pulmonary vasculature, lung parenchyma, pleura, mediastinum, and chest wall concurrently (13, 14). In addition to confirming or excluding PE, CTPA is valuable because it provides diagnostic information on other thoracic abnormalities, including consolidation, ground-glass opacities, pleural effusions, and pulmonary nodules, that may relate to the patient's presenting symptoms or represent incidental findings needing further evaluation (12, 15).

Interpretation of CTPA findings in cancer patients is particularly challenging, as those patients frequently demonstrate a variety of pleuroparenchymal abnormalities related not only to their primary tumor, but also to metastatic disease, secondary infection, and/or treatment-related lung injury, including changes from chemotherapy and/or radiation therapy (10, 16). The combined imaging findings can mimic or obscure the appearance of PE, complicating accurate interpretation of the study. Furthermore, the specific cancer type may impact both the frequency of occurrence and the type of radiographic appearance for pleuroparenchymal abnormalities identified on CTPA, creating additional challenges for the clinician in differentiating embolic from non-embolic causes of the abnormalities.

While numerous studies have evaluated pleuroparenchymal findings of patients who received CTPA for suspected PE, the majority have been conducted in the general patient population, with limited attention to those with a malignancy (17, 18). As such, there is still much more to learn about how a malignancy affects the spectrum of pleuroparenchymal abnormalities detected on CTPA, both in terms of frequency and severity, in relation to whether there is a presence or absence of PE.

Cancer-specific studies of CTPA have focused mainly on diagnostic yield and predictors of a positive examination (19), or on incidentally detected embolism (20), whereas the pleural and parenchymal content of these scans has been catalogued almost exclusively in unselected populations (17, 18). It therefore remains unclear which pleuroparenchymal abnormalities occur in cancer patients imaged for suspected PE, and which of them differ between patients with and without confirmed embolism. Accordingly, this study characterized these abnormalities in a consecutive oncologic cohort and compared them between PE-positive and PE-negative examinations, aiming to distinguish findings of discriminatory value from nonspecific background pathology.

## Methodology

### Study setting and design

We did a retrospective observational study at a tertiary referral hospital that has an extremely busy oncology and radiology department, to evaluate CTPA studies performed in cancer patients with suspected acute PE between Jan 1, 2022 and Jan 24, 2026. Out of 194 initial cases identified, 113 met the inclusion criteria. The study was designed and reported in accordance with the Strengthening the Reporting of Observational Studies in Epidemiology (STROBE) guidelines (21). The full study protocol is available upon request.

### Sampling strategy and data collection

Consecutive sampling technique was followed to minimize selection bias, where we include all eligible CTPA exams that met the inclusion criteria for this study within the time frame specified. Data was extracted from the hospital's Picture Archiving and Communication System (PACS) and electronic medical records using a pre-specified structured data sheet, developed and finalized prior to data collection. The sheet captured patient demographic characteristics (age and sex), malignancy type and stage, CTPA result with respect to PE and its distribution, and detailed pleural and pulmonary parenchymal imaging findings. Pleural and parenchymal findings were extracted from the formal clinical radiology reports issued at the time of examination, which were produced in routine practice by a consultant radiologist with more than eight years of subspecialty experience in thoracic imaging. These reports predated the present study and were generated without knowledge of its research question, so that parenchymal descriptions were not recorded in anticipation of the comparisons subsequently undertaken. No dedicated re-review of the source images was performed for the purposes of this study. Extraction followed predefined criteria specified before data collection, and findings described in equivocal terms were recorded as absent unless explicitly reported. Because a single clinical report was available per examination, interobserver agreement could not be assessed.

Imaging terminology was classified according to the definitions of the Fleischner Society glossary of terms for thoracic imaging (22), applied to the descriptions recorded in the formal radiology reports. These definitions are provided in the Supplementary Table S1.

### Inclusion and exclusion criteria

Consecutive inpatients and outpatients with clinically suspected acute (first or recurrent) PE were eligible for inclusion if they were aged 18 years or older, had a histologically or clinically confirmed malignancy (solid or hematologic), and underwent a technically adequate CTPA with good diagnostic-quality and satisfactory opacification of the pulmonary arterial tree. Patients were excluded if they had a non-diagnostic or technically inadequate CTPA, had no confirmed malignancy, had previously been included in the study based on a prior CTPA examination, or had incomplete imaging or clinical records preventing reliable extraction of study variables.

### Primary and secondary outcomes

The primary outcome was the presence, type, and distribution of pleuroparenchymal abnormalities on CTPA, with each finding characterized by extent (focal vs. diffuse), laterality (unilateral vs. bilateral), and severity (mild, moderate, or severe), based on predefined radiological criteria applied to the formal radiology report. Secondary outcomes of this study are threefold. First, we aim to evaluate the association between specific pleuroparenchymal abnormalities and the presence of PE in patients with malignancy. Second, we seek to identify characteristic CT imaging patterns that may help differentiate PE-related alterations from other pulmonary abnormalities commonly observed in cancer patients or arising as a consequence of oncologic treatment. Finally, we intend to provide baseline radiological data that can support more accurate diagnostic interpretation and inform clinical decision-making in cancer patients undergoing CTPA for suspected PE.

### Statistical analysis

Statistical analyses were performed using IBM SPSS Statistics version 27.0 (IBM Corp., Armonk, NY, USA). Continuous variables were summarized as mean ± standard deviation or median with interquartile range (IQR), as appropriate, and categorical variables as frequencies and percentages. Comparisons between patients with and without pulmonary embolism were made using the independent-samples *t* test or Mann–Whitney *U* test for continuous variables and the chi-square test or Fisher's exact test for categorical variables. Unadjusted odds ratios (ORs) with 95% confidence intervals (CIs) were calculated for imaging findings associated with pulmonary embolism. To address the limited number of PE events (*n* = 32) and reduce overfitting, the exploratory multivariable logistic regression was deliberately restricted to three clinically plausible imaging predictors with the strongest univariable signals: ground-glass opacity, interstitial/septal change, and infarct-like wedge-shaped opacity. This yielded 10.7 events per predictor; demographic and cancer-subgroup variables were not entered because the model was intended to evaluate imaging features and preserve parsimony. Multicollinearity was assessed using variance inflation factors (VIFs), with VIF <5 considered acceptable. Calibration was evaluated using the Hosmer-Lemeshow goodness-of-fit test based on the six distinct predicted-risk strata and a grouped calibration plot. Discrimination was summarized by the area under the receiver operating characteristic curve (AUROC) and internally validated using 2,000 bootstrap resamples to estimate optimism-adjusted AUROC. Benjamini–Hochberg false-discovery-rate (FDR) correction was applied across the 12 imaging comparisons reported in Table 3, with Bonferroni correction used as a sensitivity analysis. Exploratory subgroup analyses compared lung versus non-lung cancer, hematologic versus solid malignancies, and documented metastatic disease versus no documented metastasis; FDR correction was applied across the 12 subgroup comparisons. Two-sided *p* < 0.05 denoted nominal statistical significance, whereas FDR-adjusted *q* < 0.05 denoted multiplicity-adjusted significance.

### Ethical approval

Ethical approval for this study was obtained from the Institutional Review Board (IRB) of the hospital prior to data collection. Given the retrospective nature of the study and the absence of any direct patient contact or intervention, the requirement for individual informed consent was waived in accordance with applicable institutional guidelines.

## Results

A total of 113 adult cancer patients who underwent CTPA for suspected PE were included in the final analysis. Among them, 32 patients (28.3%) had radiologically confirmed PE, whereas 81 patients (71.7%) had no evidence of embolism. The median age of the overall cohort was 59 years (IQR, 51–65 years), and 48 patients (42.5%) were female. Baseline characteristics according to PE status are presented in Table 1. There was no statistically significant difference in age between the PE-positive and PE-negative groups, with median ages of 58 years (IQR, 51–63) and 59 years (IQR, 49–65), respectively (*p* = 0.891). Sex distribution was also comparable between groups, although women were less frequent in the PE-positive group than in the PE-negative group (31.2% vs. 46.9%, *p* = 0.145). Seventy-six patients (67.3%) were classified as smokers; smokers comprised 17 of 32 PE-positive patients (53.1%) and 59 of 81 PE-negative patients (72.8%; *p* = 0.044). Similarly, the distribution of primary malignancy categories did not differ significantly between groups (*p* = 0.191). Of the overall cohort, 42 patients (37.2%) had documented metastatic disease and 71 (62.8%) had no documented metastasis, with no significant difference between the PE-positive and PE-negative groups (*p* = 0.413).

**Table 1:** Baseline characteristics by pulmonary embolism status.

Characteristic	Overall (*N* = 113)	PE + (*n* = 32)	PE− (*n* = 81)	*P* value
Age, years	59 [51–65]	58 [51–63]	59 [49–65]	0.891
Female sex	48 (42.5%)	10 (31.2%)	38 (46.9%)	0.145
Smoking history	76 (67.3%)	17 (53.1%)	59 (72.8%)	0.044
Primary malignancy group				0.191
Thoracic/Lung	19 (16.8%)	6 (18.8%)	13 (16.0%)	
Breast	14 (12.4%)	2 (6.2%)	12 (14.8%)	
GI/Hepatobiliary	29 (25.7%)	10 (31.2%)	19 (23.5%)	
Hematologic	10 (8.8%)	0 (0.0%)	10 (12.3%)	
Gynecologic/GU	14 (12.4%)	6 (18.8%)	8 (9.9%)	
Other solid tumors	27 (23.9%)	8 (25.0%)	19 (23.5%)	
Metastatic status				0.413
Metastatic	42 (37.2%)	10 (31.2%)	32 (39.5%)	
No documented metastasis	71 (62.8%)	22 (68.8%)	49 (60.5%)	

Smoking counts were supplied as PE-group totals. “No documented metastasis” denotes absence of documented metastatic disease in the available stage field and should not be interpreted as confirmation of localized stage.

GI, gastrointestinal; GU, genitourinary.

Exploratory analyses by cancer subtype and metastatic status are presented in the Supplementary Table S2. PE prevalence did not differ between lung and non-lung cancer (27.8% vs. 28.4%; *p* = 1.000), hematologic and solid malignancies (0.0% vs. 31.1%; *p* = 0.060), or documented metastatic disease and no documented metastasis (23.8% vs. 31.0%; *p* = 0.518). Ground-glass opacity was more frequent in hematologic malignancy (70.0% vs. 26.2%; unadjusted *p* = 0.0078), while interstitial/septal change was more frequent in lung cancer (38.9% vs. 15.8%; *p* = 0.045); neither remained significant after FDR correction (*q* = 0.094 and *q* = 0.179, respectively). No exploratory subgroup comparison met *q* < 0.05.

The overall prevalence of pleural and parenchymal CT abnormalities is summarized in Table 2. The most common radiologic finding in the entire cohort was pleural effusion, which was present in 66 patients (58.4%). Other frequent abnormalities included pulmonary nodules or masses in 52 patients (46.0%), atelectatic or collapse changes in 51 patients (45.1%), consolidation in 43 patients (38.1%), and ground-glass opacity in 34 patients (30.1%). Interstitial or septal changes were observed in 22 patients (19.5%), while infectious or inflammatory patterns were identified in 16 patients (14.2%). Less common findings included emphysematous change in 13 patients (11.5%), bronchiectatic or tree-in-bud changes in 12 patients (10.6%), cavitary or cystic lesions in 6 patients (5.3%), and infarct-like wedge-shaped opacities in 5 patients (4.4%). Pleural thickening, pneumothorax or hydropneumothorax, and pleural nodules or masses were infrequent.

**Table 2:** Overall prevalence of pleural and parenchymal CT findings.

Finding	*n*	Prevalence
Parenchymal findings		
Atelectatic/collapse changes	51	45.1%
Consolidation	43	38.1%
Ground-glass opacity	34	30.1%
Pulmonary nodules/masses	52	46.0%
Interstitial/septal change	22	19.5%
Emphysematous change	13	11.5%
Bronchiectatic/tree-in-bud change	12	10.6%
Infectious/inflammatory pattern	16	14.2%
Cavitary/cystic lesion	6	5.3%
Infarct-like wedge-shaped opacity	5	4.4%
Pleural findings		
Pleural effusion	66	58.4%
Pleural thickening	3	2.7%
Pneumothorax/hydropneumothorax	4	3.5%
Pleural nodules/masses	1	0.9%

Comparative analysis of imaging findings according to PE status is shown in Table 3, with graphical presentation in Figures 1, 2. At the nominal unadjusted level, infarct-like wedge-shaped opacity was more frequent in PE-positive patients than in PE-negative patients (12.5% vs. 1.2%; OR, 11.43; 95% CI, 1.23–106.62; *p* = 0.022). The width of this interval reflects the small number of events on which the estimate is based, with the finding present in four PE-positive and one PE-negative patient. Ground-glass opacity (15.6% vs. 35.8%; OR, 0.33; 95% CI, 0.12–0.96; *p* = 0.041) and interstitial/septal change (3.1% vs. 25.9%; OR, 0.09; 95% CI, 0.01–0.72; *p* = 0.007) were less frequent in PE-positive patients. However, none of the 12 imaging comparisons remained statistically significant after Benjamini–Hochberg FDR correction (*q* = 0.133, 0.166, and 0.082 for wedge-shaped opacity, ground-glass opacity, and interstitial/septal change, respectively), and none met the Bonferroni threshold of *p* < 0.0042. Accordingly, all individual imaging associations are interpreted as exploratory. No nominally significant differences were observed for the remaining parenchymal or pleural findings.

**Table 3:** Imaging findings by pulmonary embolism status.

Finding	PE + (*n* = 32)	PE− (*n* = 81)	Unadjusted OR (95% CI)	Unadjusted *P*/FDR *q*
Atelectatic/collapse changes	18 (56.2%)	33 (40.7%)	1.87 (0.82–4.28)	0.148/0.444
Consolidation	10 (31.2%)	33 (40.7%)	0.66 (0.28–1.58)	0.396/0.798
Ground-glass opacity	5 (15.6%)	29 (35.8%)	0.33 (0.12–0.96)	0.041/0.166
Pulmonary nodules/masses	13 (40.6%)	39 (48.1%)	0.74 (0.32–1.69)	0.533/0.864
Interstitial/septal change	1 (3.1%)	21 (25.9%)	0.09 (0.01–0.72)	0.007/0.082
Emphysematous change	3 (9.4%)	10 (12.3%)	0.73 (0.19–2.86)	0.756/1.000
Bronchiectatic/tree-in-bud change	3 (9.4%)	9 (11.1%)	0.83 (0.21–3.28)	>0.99/1.000
Cavitary/cystic lesion	2 (6.2%)	4 (4.9%)	1.28 (0.22–7.38)	>0.99/1.000
Infarct-like wedge-shaped opacity	4 (12.5%)	1 (1.2%)	11.43 (1.23–106.62)	0.022/0.133
Pleural effusion	21 (65.6%)	45 (55.6%)	1.53 (0.65–3.58)	0.399/0.798
Pleural thickening	1 (3.1%)	2 (2.5%)	1.27 (0.11–14.56)	>0.99/1.000
Pneumothorax/hydropneumothorax	0 (0.0%)	4 (4.9%)	0.00 (0.01–5.06)	0.576/0.864

Benjamini–Hochberg FDR correction was applied across the 12 comparisons shown. No finding met *q* < 0.05. The Bonferroni-corrected threshold was *p* < 0.0042.

**Figure 1:**
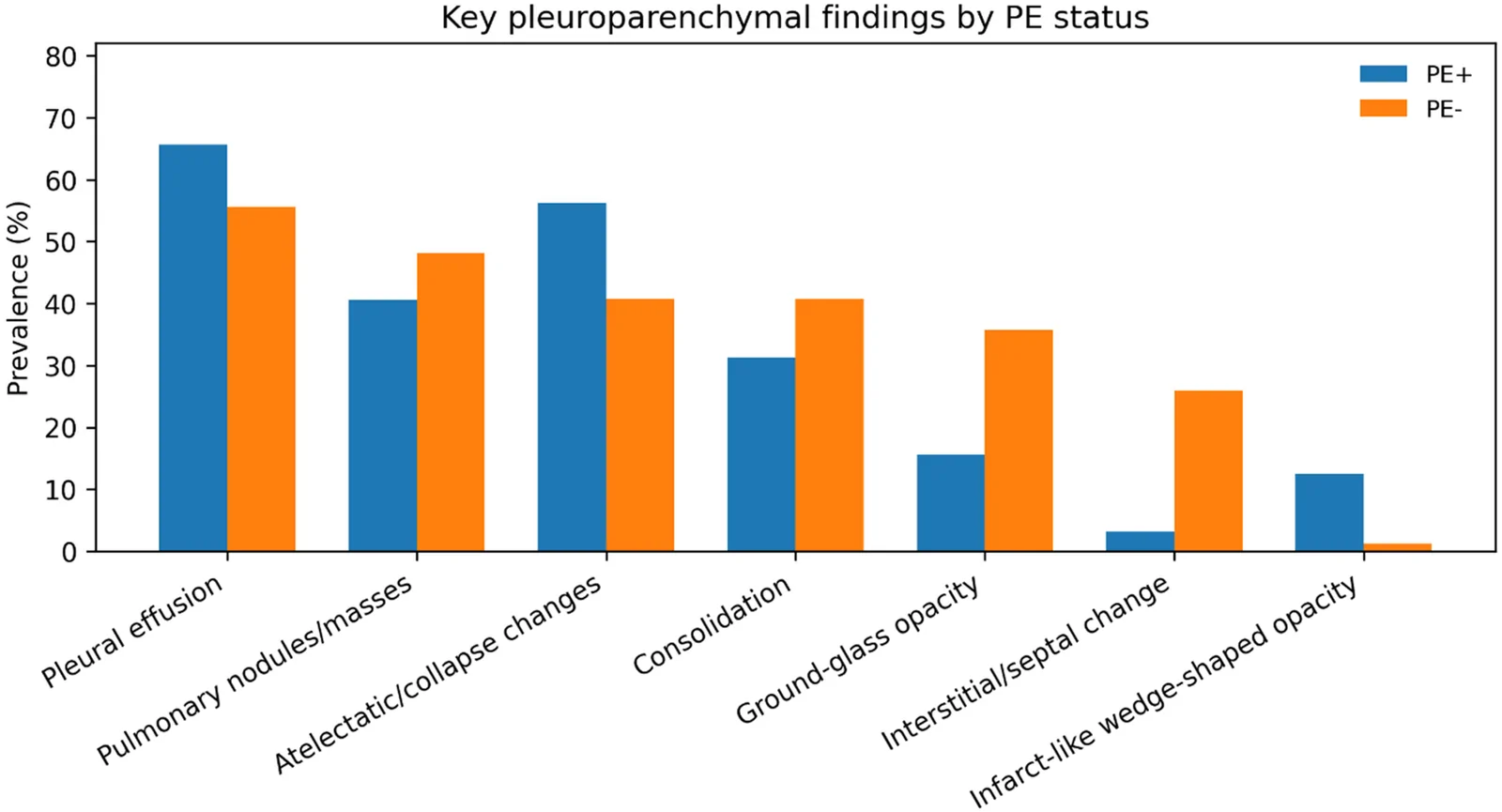
Frequency of key pleural and parenchymal CT findings according to pulmonary embolism status.

**Figure 2:**
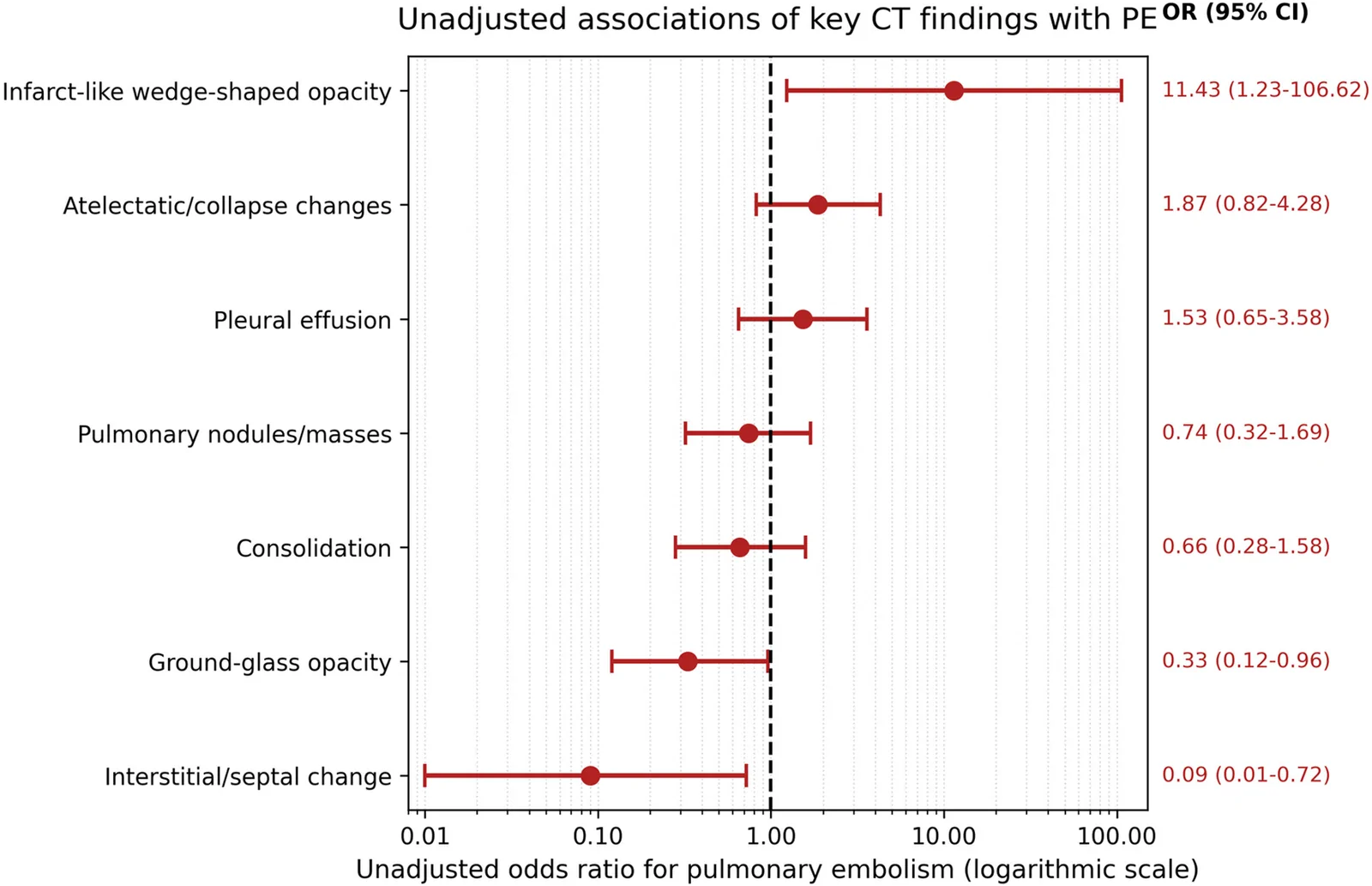
Forest plot of unadjusted odds ratios for key imaging findings associated with pulmonary embolism. The *x*-axis is logarithmic, the dashed vertical line denotes OR = 1, and horizontal bars show 95% confidence intervals.

To reduce overfitting, the exploratory multivariable model was limited to ground-glass opacity, interstitial/septal change, and infarct-like wedge-shaped opacity (32 PE events; 10.7 events per predictor), as shown in Table 4. Infarct-like wedge-shaped opacity retained a positive association with PE (adjusted OR [aOR], 11.72; 95% CI, 1.13–121.14; *p* = 0.039), whereas ground-glass opacity (aOR, 0.35; 95% CI, 0.11–1.15; *p* = 0.085) and interstitial/septal change (aOR, 0.13; 95% CI, 0.02–1.06; *p* = 0.057) did not reach nominal significance. VIFs ranged from 1.02 to 1.09, providing no evidence of problematic multicollinearity. The model was statistically significant (likelihood-ratio *p* = 0.00046; McFadden pseudo-R^2^ = 0.133) and had an apparent AUROC of 0.707 (bootstrap 95% CI, 0.618–0.788). Across 2,000 bootstrap resamples, mean optimism was 0.009, yielding an optimism-adjusted AUROC of 0.699. Grouped calibration was acceptable (Hosmer-Lemeshow *χ*^2^ = 3.22, df = 4, *p* = 0.522), although the upper predicted-risk groups were small; the calibration plot is shown in Supplementary Figure S1. Given the sparse imaging patterns and wide confidence intervals, this model remains exploratory rather than a validated clinical prediction tool.

**Table 4:** Parsimonious exploratory multivariable logistic regression for pulmonary embolism.

Predictor	Adjusted OR (95% CI)	*P* value	VIF
Ground-glass opacity	0.35 (0.11–1.15)	0.0850	1.08
Interstitial/septal change	0.13 (0.02–1.06)	0.0571	1.09
Infarct-like wedge-shaped opacity	11.72 (1.13–121.14)	0.0389	1.02

Model metrics: 32 events; 10.7 events/predictor; likelihood-ratio *p* = 0.00046; McFadden pseudo-*R*^2^ = 0.133; Brier score=0.172; AUROC=0.707 (bootstrap 95% CI, 0.618–0.788); mean bootstrap optimism=0.009; optimism-adjusted AUROC=0.699; Hosmer–Lemeshow *χ*^2^(4) = 3.22, *p* = 0.522; 2,000 bootstrap resamples.

Among the 32 patients with confirmed PE, 23 patients (71.9%) had bilateral emboli and 9 patients (28.1%) had unilateral emboli. The anatomic distribution of emboli is illustrated in Figure 3. Central PE limited to the main or lobar pulmonary arteries was observed in 9 patients (28.1%), combined central and peripheral PE in 7 patients (21.9%), segmental-only PE in 6 patients (18.8%), and mixed peripheral PE in 10 patients (31.2%). Representative CTPA images demonstrating a segmental filling defect and its associated peripheral wedge-shaped consolidation consistent with pulmonary infarction in the same patient are presented in Figures 4, 5. To further illustrate the comparative imaging findings, a representative PE-negative CTPA examination demonstrating ground-glass opacity and interstitial/septal change is presented in Figure 6.

**Figure 3:**
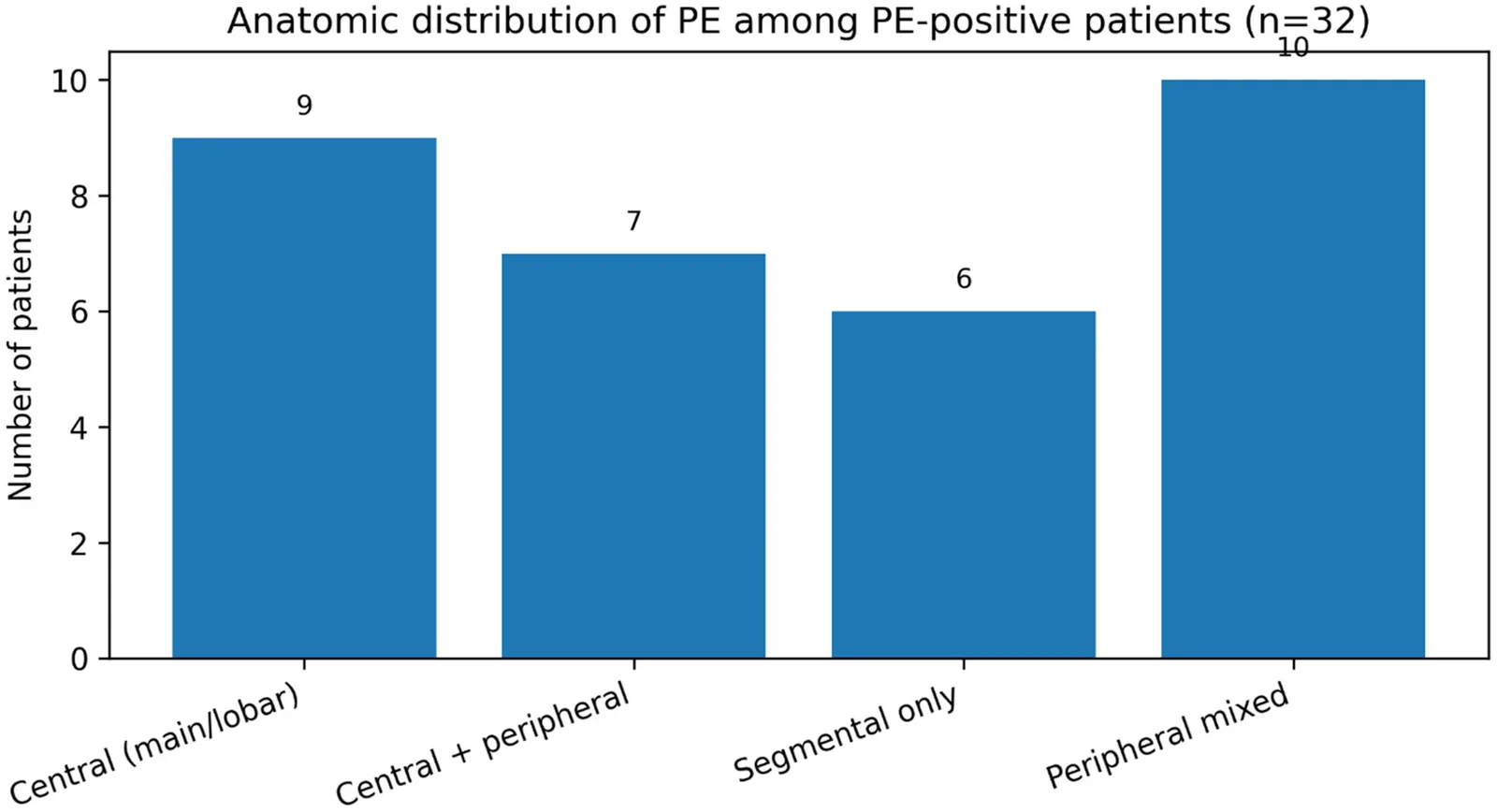
Distribution of PE laterality and anatomic level among PE-positive patients.

**Figure 4:**
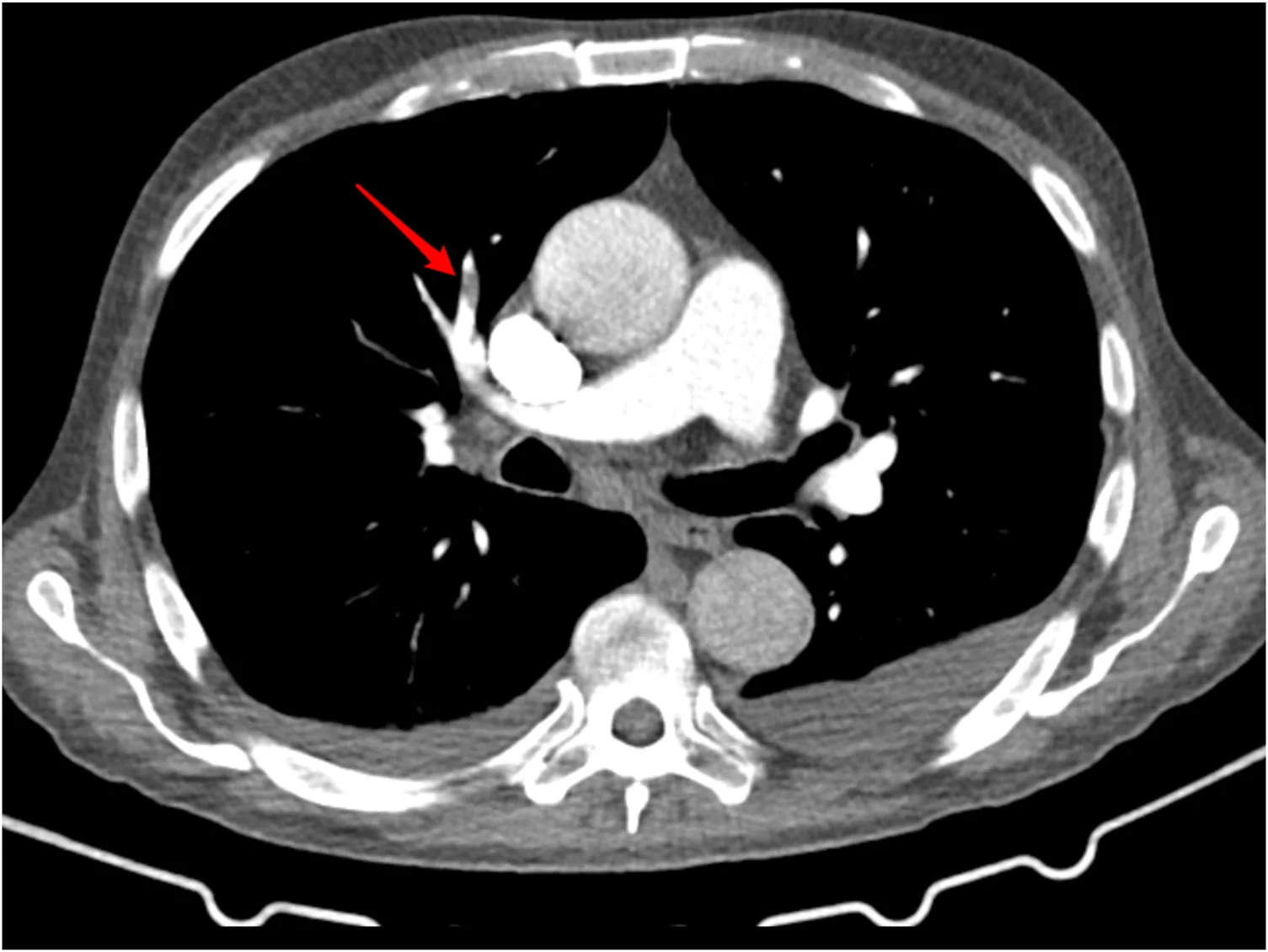
Axial contrast-enhanced CTPA of a 60-year-old male with sigmoid colon adenocarcinoma demonstrates a hypodense intraluminal filling defect (red arrow) within the segmental pulmonary artery of the right middle lobe (RML), consistent with acute PE.

**Figure 5:**
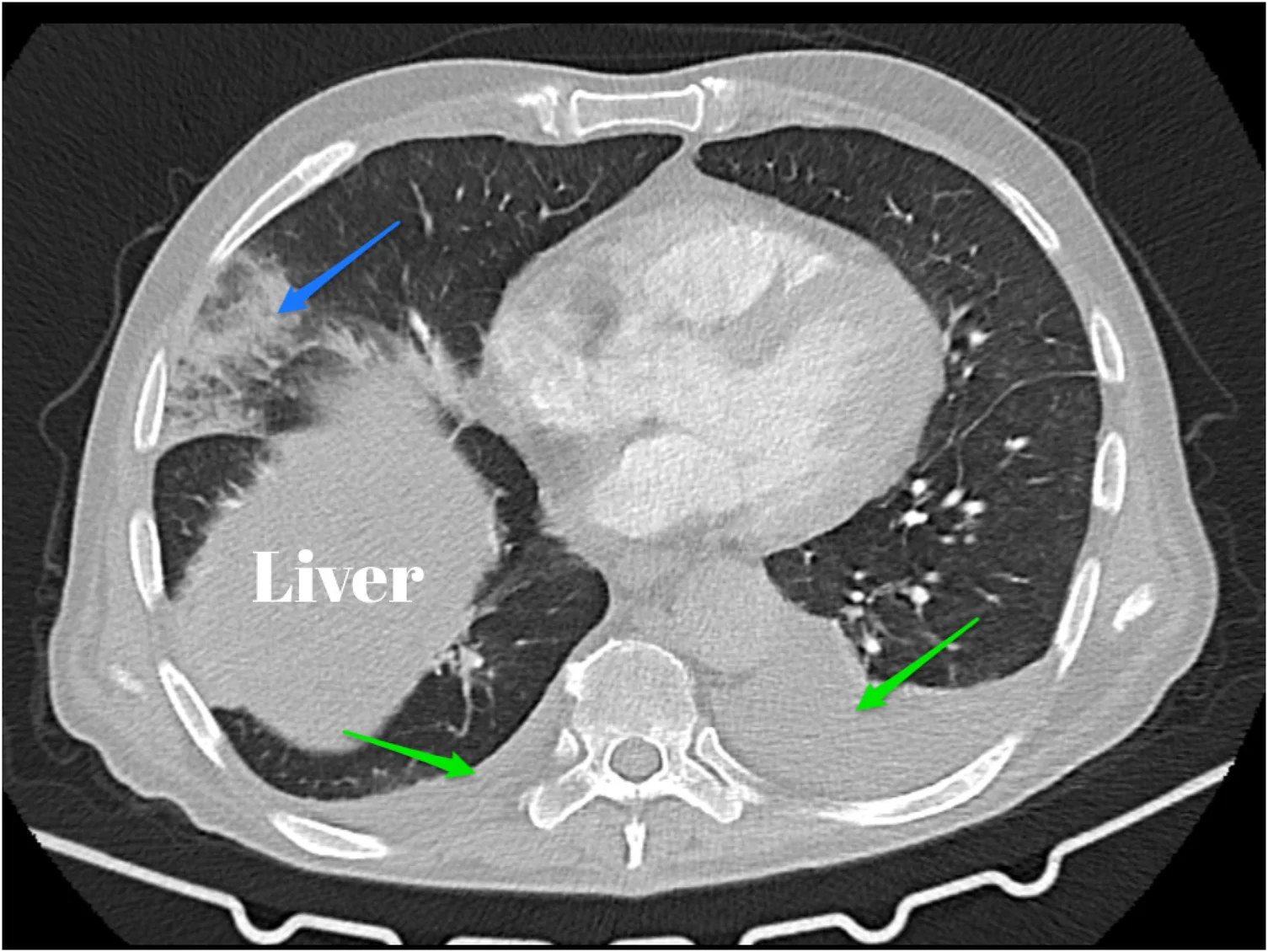
Axial CTPA image of the same patient illustrated in Figure 4 demonstrates a subpleural wedge-shaped area of consolidation in the RML with its base opposing the visceral pleural surface (blue arrow), in keeping with pulmonary infarction as a downstream parenchymal sequela of segmental arterial occlusion. Bilateral pleural effusions are also noted (green arrows), more pronounced on the left.

**Figure 6:**
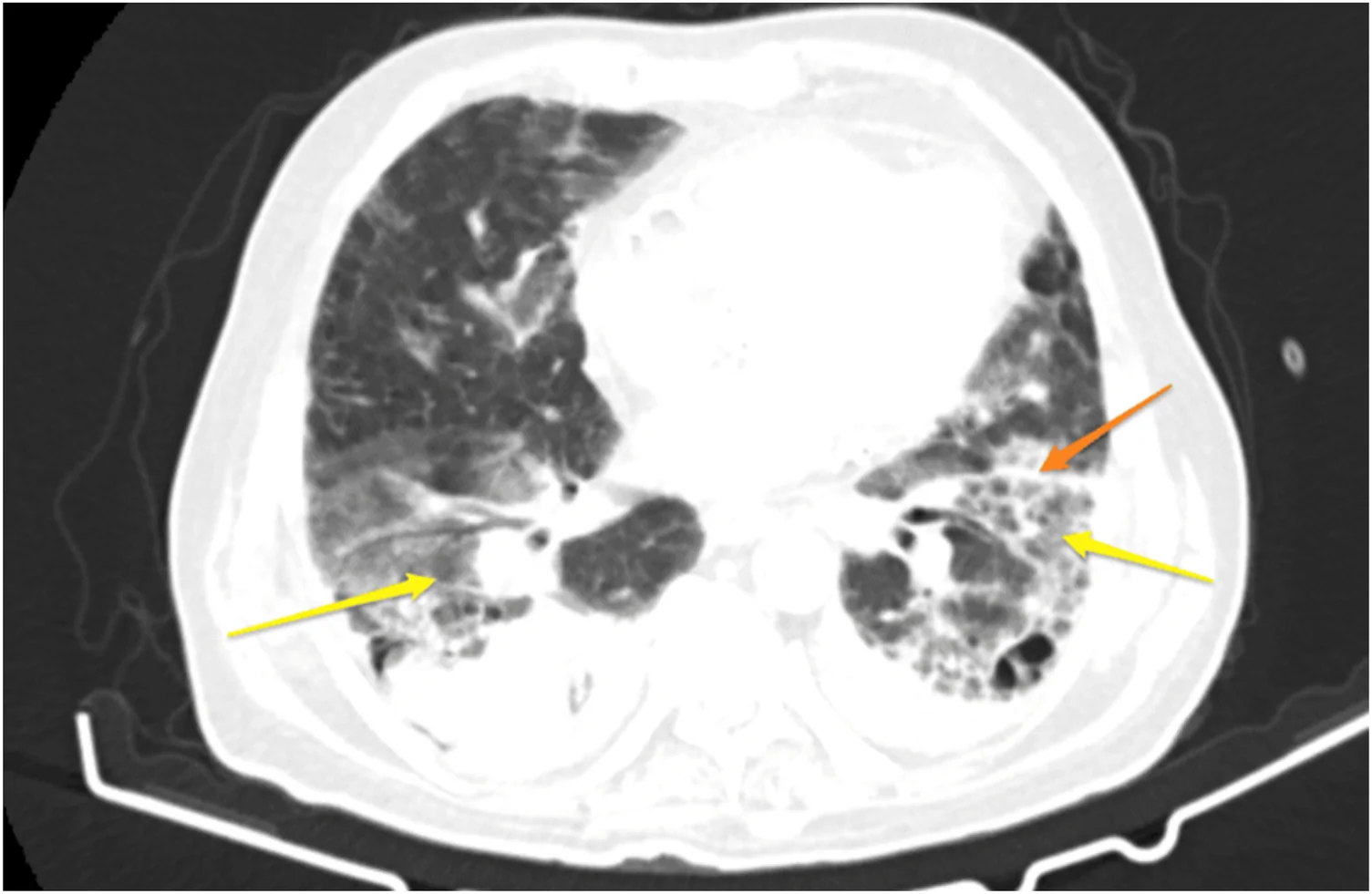
Axial contrast-enhanced PE-negative CTPA examination of a 76-year-old male with localized lung cancer demonstrates extensive bilateral ground-glass opacities (yellow arrows) and interlobular septal thickening (orange arrow), with mild bilateral pleural effusion.

## Discussion

In this retrospective observational cohort of 113 adult cancer patients who underwent CTPA for suspected PE, radiologically confirmed PE was identified in 32 patients (28.3%), underscoring the substantial thromboembolic burden within this oncologic population. The principal finding of the present study is that pleuroparenchymal abnormalities were highly prevalent overall, yet most lacked discriminatory value for PE. Against this nonspecific radiologic background, only a limited number of imaging patterns appeared to meaningfully distinguish PE-positive from PE-negative examinations. Because none of the individual imaging comparisons survived FDR or Bonferroni correction, the observed between-group differences should be interpreted as hypothesis-generating rather than confirmatory.

Across the full cohort, pleural effusion was the most frequent thoracic abnormality (58.4%), followed by pulmonary nodules or masses (46.0%), atelectatic or collapse changes (45.1%), consolidation (38.1%), and ground-glass opacity (30.1%). Additional abnormalities, including interstitial or septal change, infectious or inflammatory patterns, emphysematous change, and bronchiectatic or tree-in-bud abnormalities, were also present in a nontrivial proportion of examinations. These data indicate that, in oncologic patients undergoing CTPA, the thorax is rarely radiologically “clean”; rather, imaging is frequently characterized by a complex superimposition of malignant, treatment-related, infectious, and chronic structural lung abnormalities that can obscure, mimic, or coexist with embolic disease (23). The diagnostic challenge in this population is not merely the identification of abnormalities, but the determination of which abnormalities are plausibly attributable to acute PE and which instead represent the broader background pathology of malignancy.

Among the pleuroparenchymal abnormalities evaluated, infarct-like wedge-shaped opacity produced the strongest exploratory signal for PE. It occurred in 12.5% of PE-positive examinations compared with 1.2% of PE-negative examinations, corresponding to an unadjusted OR of 11.43. In the parsimonious three-predictor model, the direction and magnitude remained positive (aOR, 11.72; 95% CI, 1.13–121.14; *p* = 0.039). However, the unadjusted comparison did not remain significant after FDR correction (*q* = 0.133), and only five patients had this finding. The extremely wide confidence intervals therefore indicate substantial imprecision, and the result should be regarded as an exploratory radiologic signal requiring independent validation rather than as evidence of a stable standalone predictor.

From a pathophysiologic perspective, this association is biologically plausible. Peripheral wedge-shaped opacities are classically linked to pulmonary infarction secondary to vascular occlusion, and in the setting of acute PE may therefore provide an important radiologic clue that the embolus is not merely intravascular but has already produced downstream parenchymal consequences (24). In cancer patients, however, even this pattern must be interpreted cautiously, as peripheral consolidative or wedge-like abnormalities may occasionally overlap with infection, hemorrhage, organizing pneumonia, or tumor-related change. Accordingly, the present findings do not suggest that wedge-shaped opacity is a sensitive marker of PE, since it was present in only a minority of PE-positive examinations, but rather that when it is identified on CTPA in an oncology patient with compatible clinical suspicion, its presence should heighten consideration of embolic pulmonary infarction.

Comparison with earlier oncology-focused imaging studies is not straightforward, as these have generally sought different objectives. Tannous and colleagues (19) reported pulmonary embolism in only 5.7% of 208 cancer patients undergoing CTPA in an emergency department, considerably below the 28.3% observed in our cohort. We consider this more likely to reflect differences in referral pattern and clinical setting than a true difference in embolic risk, as our patients were recruited from a tertiary hospital with a high-volume oncology service rather than from an emergency department population; our observed prevalence is, however, consistent with the diagnostic yield of approximately 23% reported among unselected patients undergoing CTPA for suspected pulmonary embolism (18). A comparable observation applies to infarct-like wedge-shaped opacity, which we identified in 12.5% of PE-positive patients, whereas pulmonary infarction, defined by established radiological criteria, was diagnosed on CT in 31% of patients in a prospective multicenter series of acute pulmonary embolism (25). Part of this difference is likely methodological, since our findings were derived from formal radiology reports rather than from dedicated re-review, and part may be attributable to the complex parenchymal background of malignancy, in which a peripheral wedge-shaped opacity may reasonably be assigned to tumor, infection, or treatment effect. Taken together, these comparisons indicate that the discriminatory value of pleuroparenchymal findings established in general populations should not be assumed to apply unchanged to patients with cancer.

At nominal unadjusted testing, ground-glass opacity and interstitial/septal change were less frequent in PE-positive than PE-negative examinations. Their FDR-adjusted values were *q* = 0.166 and *q* = 0.082, respectively, and neither met the prespecified *q* < 0.05 criterion. In the reduced multivariable model, both estimates remained inverse, but ground-glass opacity (aOR, 0.35; *p* = 0.085) and interstitial/septal change (aOR, 0.13; *p* = 0.057) did not reach nominal significance. These patterns remain clinically plausible indicators of competing oncologic, infectious, edematous, or treatment-related thoracic processes, but the present data do not establish independent protective or exclusionary effects for PE.

From a clinical perspective, this pattern is highly plausible in the oncologic setting. Ground-glass and interstitial or septal abnormalities are radiologically heterogeneous and may reflect a broad spectrum of alternative or coexisting disease processes, including infection, pulmonary edema, lymphangitic tumor spread, drug-related pneumonitis, radiation-induced injury, or other inflammatory and treatment-related parenchymal changes (26). In cancer patients, where such entities are common and may coexist with symptoms that overlap substantially with those of PE, these findings are unlikely to serve as positive radiologic markers of embolic disease (20, 27). Rather, their relative predominance in PE-negative examinations suggests that they should prompt careful consideration of non-PE explanations for respiratory symptoms, even when embolism remains part of the initial differential diagnosis (6). The present design cannot establish that these patterns exclude PE, and an inverse association observed in a cohort selected on clinical suspicion may partly reflect referral behavior, since patients with ground-glass or interstitial abnormalities may undergo CTPA precisely because those findings generate dyspnea attributable to non-embolic disease. Embolism must therefore continue to be assessed on the basis of direct vascular findings irrespective of the surrounding parenchymal pattern.

The main mechanism likely lies in the radiological specificity of embolic parenchymal injury. Acute pulmonary embolism produces focal, peripheral change reflecting segmental vascular occlusion with distal infarction or regional oligaemia, rather than the diffuse alveolar or interlobular septal abnormalities that define ground-glass opacity and interstitial thickening (24). Embolism would therefore not be expected to generate these patterns, whose presence points instead to processes affecting the alveolar–interstitial compartment more diffusely (16, 26).

Among PE-positive patients, bilateral embolic involvement was documented in 71.9%, with emboli distributed across central, segmental, and mixed peripheral vascular territories. Taken together, these data suggest that, in this oncologic cohort, PE was frequently not a small isolated finding but rather a bilateral and often anatomically extensive thromboembolic process. This pattern carries two important clinical implications. First, it reinforces the concept that cancer-associated thromboembolic disease may present with a substantial clot burden, which may in turn increase the likelihood of downstream parenchymal consequences. Second, the predominance of bilateral and mixed-distribution emboli highlights the need for comprehensive scrutiny of the entire pulmonary arterial tree when reviewing CTPA in oncology patients, particularly because the surrounding thoracic background is often dominated by coexisting abnormalities. Although the current study was not designed to correlate embolic distribution with clinical severity or outcomes, the observed predominance of bilateral and anatomically widespread PE supports the view that thromboembolic disease in cancer patients should be approached as a potentially extensive process rather than being incidental or minor.

The present findings have several practical implications for image interpretation and bedside decision-making in oncology patients undergoing CTPA for suspected PE. First, although none of the pleuroparenchymal findings described here is diagnostic of pulmonary embolism, these findings should be interpreted in the context of vascular assessment. The diagnosis rests on direct demonstration of an intravascular filling defect within the pulmonary arterial tree, and the patterns examined in the present study can at most modify the index of suspicion once that vascular assessment has been completed. With this caveat in mind, our results support a deliberately context-sensitive approach to thoracic CT interpretation in this population. Because cancer patients frequently demonstrate a high background complexity, the mere presence of pleuropulmonary findings should not be assumed to confirm or refute PE in isolation (28, 29). Rather, any interpretive value lies in the pattern of abnormalities and their relationship to the vascular findings.

Second, the data are consistent with reading CTPA in oncology as a dual-purpose examination, a vascular study for emboli and a comprehensive thoracic study for alternative or coexisting causes of symptoms. Clinically, the results therefore support a reporting model in which radiologists clearly separate: (i) direct vascular evidence of PE, (ii) parenchymal findings plausibly related to embolic injury, and (iii) ancillary abnormalities more suggestive of nonembolic thoracic disease. Such an approach may improve communication with treating clinicians, reduce overattribution of nonspecific abnormalities to PE, and better align with the original study aim of improving radiologic interpretation and clinical decision-making in cancer patients evaluated for suspected embolism (30, 31).

The present study has several notable strengths. First, it addresses a clinically relevant and comparatively underexplored question in a distinctly high-risk population, in whom image interpretation is frequently complicated by overlapping thoracic abnormalities. In this respect, the study extends the discussion beyond embolus detection alone and instead evaluates the broader pleuroparenchymal context in which CTPA is interpreted in routine oncology practice. Second, the study design captures a real-world consecutive hospital-based cohort, which enhances the practical relevance of the findings. The inclusion of both PE-positive and PE-negative patients within the same clinical pathway is a particular strength, as it allows direct comparison of imaging patterns across the two diagnostically relevant groups rather than describing abnormalities in isolation.

Third, the study used a structured data abstraction approach with predefined imaging variables, prospectively specified outcome definitions, and standardized extraction from radiology records, with anonymization, random cross-checking against original files, and adherence to STROBE reporting principles. These features strengthen methodological transparency and help reduce avoidable information bias in a retrospective setting. Finally, the analysis provides both descriptive and inferential value. In addition to documenting the overall prevalence of pleural and parenchymal abnormalities, the study identifies a limited number of imaging patterns that appear meaningfully associated with PE, and it further tests those signals in an exploratory multivariable model.

Several limitations should be acknowledged. First, the retrospective single-center design inherently limits causal inference and constrains generalizability, as it reflects the case mix, imaging workflow, and reporting practices of one center. The absolute number of PE events remained modest, which is particularly relevant for less frequent CT findings such as infarct-like wedge-shaped opacity, occurring in only five patients overall. The wide confidence intervals observed for several odds ratios therefore indicate limited precision and suggest that some effect estimates, although clinically suggestive, should be interpreted cautiously. The multivariable model should likewise be regarded as exploratory rather than definitive. Third, while malignancy type and stage were recorded, the current analysis could not fully account for potentially important confounders such as recent chemotherapy or immunotherapy, thoracic radiotherapy, active pulmonary infection, pre-existing chronic lung disease, anticoagulant use, D-dimer concentration, and performance status. These data were not consistently documented in the retrospective records available to us. Finally, as in many retrospective imaging studies, the study cannot fully disentangle whether individual pleuroparenchymal findings were directly attributable to PE or other overlapping processes occurring simultaneously. Accordingly, the present results should be understood as pattern-based associations within a complex clinical-radiologic environment rather than as proof of exclusive pathophysiologic attribution. The revised model was restricted to three predictors, yielding 10.7 events per predictor, and showed low VIFs, acceptable grouped calibration, and only a small reduction from apparent to optimism-adjusted AUROC (0.707–0.699). Nevertheless, rare predictor patterns—especially wedge-shaped opacity—and very wide confidence intervals mean that residual overfitting and instability cannot be excluded. None of the 12 imaging comparisons or 12 exploratory subgroup comparisons met FDR *q* < 0.05, so nominal *p* values should not be interpreted as confirmatory. Cancer-stage information was frequently undocumented; consequently, the subgroup analysis contrasts documented metastatic disease with no documented metastasis rather than confirmed metastatic versus localized disease. In addition, the smoking reanalysis was limited to the supplied group totals and could not be linked to individual patient records for multivariable adjustment.

Future research should build on these findings through larger, multicenter studies with expanded sample size designed to validate the observed imaging associations across more diverse oncology populations and institutional settings. Such studies would be particularly valuable for confirming whether infarct-like wedge-shaped opacity consistently represents the most PE-suggestive pleuroparenchymal pattern in cancer patients. Prospective designs would further strengthen the field by enabling more detailed and standardized characterization of imaging findings. In particular, future studies should incorporate dedicated blinded radiologic re-review, formal interobserver agreement analysis, and explicit severity or extent scoring systems for pleural and parenchymal abnormalities. Future work should examine whether the relationship between pleuroparenchymal abnormalities and PE differs according to cancer subtype, metastatic burden, thoracic versus non-thoracic primary disease, recent chemotherapy, radiotherapy exposure, immunosuppression, or infectious status. Similarly, correlating CT patterns with laboratory data, symptom profiles, clot burden, treatment decisions, and clinical outcomes would help determine whether these radiologic findings are not only diagnostically informative but also prognostically relevant. Such work would move the field beyond descriptive imaging prevalence toward clinically actionable phenotyping.

## Conclusion

Among cancer patients undergoing CT pulmonary angiography for suspected pulmonary embolism, pleuroparenchymal abnormalities were highly prevalent but were largely nonspecific. Within this complex oncologic imaging background, infarct-like wedge-shaped opacity generated the strongest exploratory PE-associated signal, whereas ground-glass opacity and interstitial/septal change were more frequent in PE-negative examinations; however, none of the unadjusted imaging comparisons survived multiplicity correction. The parsimonious internally validated model showed only modest discrimination and highly imprecise coefficients. These findings support careful contextual interpretation of CTPA as both a vascular and comprehensive thoracic examination, while underscoring the need for larger multicenter external validation before any imaging pattern is used as an independent diagnostic discriminator in oncology patients.

## Data Availability

The original contributions presented in the study are included in the article/Supplementary Material, further inquiries can be directed to the corresponding author.
