# Supplementary material for: Pleuroparenchymal abnormalities in cancer patients undergoing CTPA for suspected pulmonary embolism
Source: Front Radiol. 2026 Oct 13;6:1901919. doi: 10.3389/fradi.2026.1901919 (PMC13710350; doi:10.3389/fradi.2026.1901919)
Supplement: Supplementary file 1 [file Datasheet1.pdf]

Supplementary Table S1. Definitions of pleural and parenchymal CT findings.

Definitions are based on the Fleischner Society glossary of terms for thoracic imaging [1], applied to descriptions recorded in the formal clinical radiology reports. Infectious/inflammatory pattern is not a Fleischner term and was defined operationally for the purposes of this study.

Finding	Definition
**Ground-glass opacity**	Hazy increased lung attenuation with preservation of bronchial and vascular margins
**Consolidation**	Homogeneous increase in parenchymal attenuation that obscures the margins of underlying vessels, with or without air bronchograms
**Interstitial/septal change**	Thickening of interlobular septa or intralobular lines producing a reticular pattern
**Atelectatic/collapse change**	Reduced inflation of all or part of the lung, with increased attenuation and displacement of fissures, vessels, or bronchi
**Pulmonary nodule/mass**	Rounded or irregular opacity measuring ≤3 cm (nodule) or >3 cm (mass)
**Emphysematous change**	Focal areas of low attenuation, usually without definable walls
**Bronchiectatic/tree-in-bud change**	Bronchial dilatation relative to the accompanying pulmonary artery with lack of normal tapering, or centrilobular branching nodular opacities
**Cavitary/cystic lesion**	Gas-filled space within an area of consolidation, a mass, or a nodule; or a round parenchymal lucency bounded by a thin wall
**Infarct-like wedge-shaped opacity**	Peripheral, pleural-based, wedge-shaped or hemispherical consolidation with its base abutting the visceral pleural surface
**Infectious/inflammatory pattern**	Composite category applied when the report described consolidation with air bronchograms, centrilobular nodularity, or tree-in-bud change and explicitly attributed these appearances to infection or inflammation
**Pleural effusion**	Fluid attenuation within the pleural space
**Pleural thickening**	Increased soft-tissue attenuation of the pleural surface
**Pneumothorax/hydropneumothorax**	Gas within the pleural space, with or without accompanying pleural fluid
**Pleural nodule/mass**	Discrete soft-tissue lesion arising from the pleural surface

Supplementary Table S2. Exploratory cancer-subgroup analyses of pulmonary embolism and key CT findings.

Contrast / outcome	First group	Comparator	OR (95% CI)	P	FDR q
Lung vs non-lung: PE *	5/18 (27.8%)	27/95 (28.4%)	0.97 (0.31–2.98)	>0.99	1.000
Lung vs non-lung: Ground-glass opacity	9/18 (50.0%)	25/95 (26.3%)	2.80 (1.00–7.85)	0.0540	0.179
Lung vs non-lung: Interstitial/septal change	7/18 (38.9%)	15/95 (15.8%)	3.39 (1.13–10.16)	0.0454	0.179
Lung vs non-lung: Wedge-shaped opacity	1/18 (5.6%)	4/95 (4.2%)	1.34 (0.14–12.72)	0.5872	1.000
Hematologic vs solid: PE	0/10 (0.0%)	32/103 (31.1%)	NE	0.0597	0.179
Hematologic vs solid: Ground-glass opacity	7/10 (70.0%)	27/103 (26.2%)	6.57 (1.58–27.23)	0.0078	0.094
Hematologic vs solid: Interstitial/septal change	1/10 (10.0%)	21/103 (20.4%)	0.43 (0.05–3.62)	0.6838	1.000
Hematologic vs solid: Wedge-shaped opacity	0/10 (0.0%)	5/103 (4.9%)	NE	>0.99	1.000
Metastatic vs no documented metastasis: PE	10/42 (23.8%)	22/71 (31.0%)	0.70 (0.29–1.66)	0.5182	1.000
Metastatic vs no documented metastasis: Ground-glass opacity	12/42 (28.6%)	22/71 (31.0%)	0.89 (0.39–2.06)	0.8347	1.000
Metastatic vs no documented metastasis: Interstitial/septal change	8/42 (19.0%)	14/71 (19.7%)	0.96 (0.36–2.52)	>0.99	1.000
Metastatic vs no documented metastasis: Wedge-shaped opacity	0/42 (0.0%)	5/71 (7.0%)	NE	0.1554	0.373

*Note. Fisher exact tests were used. FDR q values were calculated across the 12 exploratory subgroup comparisons. NE indicates that the odds ratio was not estimable because of a zero cell. The metastatic comparison contrasts documented metastatic disease with no documented metastasis, not confirmed metastatic versus localized stage. * Lung cancer subgroup analyses were restricted to patients with a primary lung malignancy (n=18) and exclude one patient with a non-lung thoracic malignancy included under "Thoracic/Lung" in Table 1 of the main manuscript.*

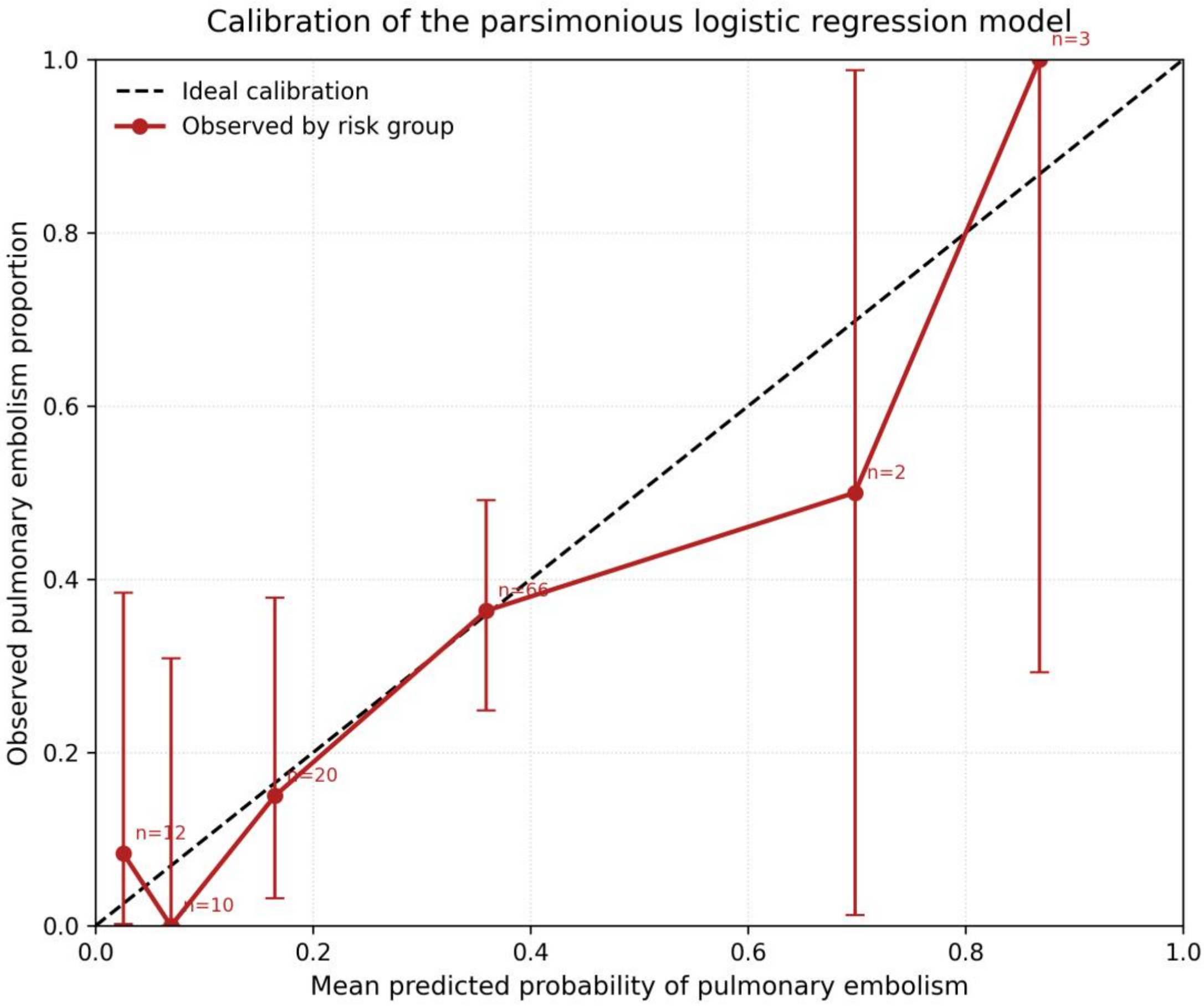

Supplementary Figure S1. Calibration plot for the parsimonious logistic regression model. Points represent the six distinct predicted-risk groups, vertical bars are exact 95% binomial confidence intervals, and the dashed line indicates perfect calibration.

References:

1. Bankier, A. A., MacMahon, H., Colby, T., Gevenois, P. A., Goo, J. M., Leung, A. N. C., Lynch, D. A., Schaefer-Prokop, C. M., Tomiyama, N., Travis, W. D., Verschakelen, J. A., White, C. S., & Naidich, D. P. (2024). Fleischner Society: Glossary of Terms for Thoracic Imaging. Radiology, 310(2), e232558. https://doi.org/10.1148/radiol.232558
